# Quantitative assessment of intraneural vascular alterations in peripheral nerve trauma using high-resolution neurosonography: technical note

**DOI:** 10.1038/s41598-021-92643-9

**Published:** 2021-06-25

**Authors:** Patrick Dömer, Ulrike Janssen-Bienhold, Bettina Kewitz, Thomas Kretschmer, Christian Heinen

**Affiliations:** 1 Department of Neurosurgery, Evangelisches Krankenhaus, Campus Carl von Ossietzky University Oldenburg, Carl von Ossietzky Str. 9-11, 26129 Oldenburg, Germany; 2 Department of Neuroscience, Carl von Ossietzky University Oldenburg, Oldenburg, Germany; 3 Research Center Neurosensory Science, Carl von Ossietzky University Oldenburg, Oldenburg, Germany; 4 Department of Neurosurgery and Neurorestauration, Klinikum Klagenfurt am Wörthersee, Klagenfurt, Austria; 5 Department of Neuro-, Spine and Nerve Surgery, Christliches Krankenhaus Quakenbrück GmbH, Quakenbrück, Germany

**Keywords:** Peripheral nervous system, Peripheral nervous system, Regeneration and repair in the nervous system

## Abstract

High-resolution neurosonography (HRNS) has become a major imaging modality in assessment of peripheral nerve trauma in the recent years. However, the vascular changes of traumatic lesions have not been quantitatively assessed in HRNS. Here, we describe the vascular-ratio, a novel HRNS-based quantitative parameter for the assessment of intraneural vascular alterations in patients with nerve lesions. N = 9 patients suffering from peripheral nerve trauma were examined clinically, electrophysiologically and with HRNS (SonoSite Exporte, Fuji). Image analyses using Fiji included determination of the established fascicular ratio (FR), the cross-section ratio (CSR), and as an extension, the calculation of a vascular ratio (VR) of the healthy versus damaged nerve and a muscle perfusion ratio (MPR) in comparison to a healthy control group. The mean VR in the healthy part of the affected nerve (14.14%) differed significantly (p < 0.0001) from the damaged part (VR of 43.26%). This coincides with significant differences in the FR and CSR calculated for the damaged part versus the healthy part and the controls. In comparison, there was no difference between VRs determined for the healthy part of the affected nerve and the healthy controls (14.14% / 17.72%). However, the MPR of denervated muscles was significantly decreased compared to the non-affected contralateral controls. VR and MPR serve as additional tools in assessing peripheral nerve trauma. Image analysis and calculation are feasible. Combined with the more morphologic FR and CSR, the VR and MPR provide a more detailed insight into alterations accompanying nerve trauma.

## Introduction

High resolution neurosonography (HRNS) has become a key factor in assessment of peripheral nerve trauma and influences decision-making in the treatment of the affected patients. Once the severity of a lesion is detected, individualized treatment can be scheduled reaching from conservative to surgical therapy. Most publications regarding HRNS deal with morphological changes and are mainly descriptive. In previous studies, a HRNS classification in accordance with the well-established Sunderland/Millesi criteria for nerve damage was developed^1^. Complementary, the so called fascicular ratio (FR) was introduced for quantification of nerve damage^2^, based on the findings of Tagliafico et al. (2014) and Boom et al. (2012). The FR allows for reliable differentiation between healthy and lesioned nerves. Moreover, the FR of a recovering nerve significantly changed back from initial pathologic to physiological values associated with a clinical and/or electrophysiological reinnervation^2^. Unfortunately, FR cannot visualize the actual process of axonal regrowth.

The vascular system seems to play an important role in nerve de- and regeneration after nerve trauma. As stated by Cattin et al. (2015), the axonal regrowth depends on a 3D scaffold of macrophage-induced blood vessels which bridges the gap between the proximal and distal nerve ends and supports the formation of Schwann cell cords for the outgrowing axons^3^.

Thus, the depiction of vascularization after nerve trauma appears to be a valuable target for a more detailed assessment of the human posttraumatic nerve using Doppler ultrasound.

## Methods

### Ethical statement

All experimental protocols were approved by the local ethics committee (“Ethik-Kommission der Carl von Ossietzky Universität Oldenburg” Drs. 49/2012). We confirm that all methods were performed in accordance with the relevant guidelines and regulations. An informed consent was obtained from all the patients or from a parent and/or legal guardian for subjects under 18 years of age.

### Patients

During 2018 and 2019, n = 9 patients suffering from traumatic nerve injury were included in this study. We retrospectively reviewed the medical charts (for clinical/demographical data, see Table 1).

**Table 1:** Synopsis of the demographic and clinical patient data.

Patient	Age (years)	Sex	Nerve	Type of injury	Neuroma type	Pain	Trauma-HRNS (weeks)	Surgery	Type of surgery	Graft length (cm)
1	12	m	Ulnar	Stretch	Continuity	Yes	4	No		
2	40	m	Median	Gun shot	Partial discontinuity	Yes	140	Yes	Split repair	8.5
3	33	m	Radial	Iatrogenic	Continuity	No	6	Yes	Complete autologous transplantation	4
4	19	m	Ulnar	Traumatic stretch	Continuity	No	12	no		
5	22	m	Peroneal	Traumatic stretch	Continuity	No	16	Yes	Complete autologous transplantation	14
6	25	m	Peroneal	Iatrogenic stretch	Continuity	No	28	Yes	Complete autologous transplantation	14
7	25	m	Peroneal	Traumatic stretch	Discontinuity	No	148	Yes	Complete autologous transplantation	14
8	71	f	Radial	Traumatic	Continuity	No	3	No		
9	25	m	Ulnar	Laceration	Partial discontinuity	No	120	Yes	Split repair	11

### HRNS

All neuro- and myosonography was performed preoperatively by an experienced neurosurgeon/neurosonologist using a 5–16 MHz ultrasound probe (SonoSite X-Porte; FujiFilm Inc, Tokyo, Japan). For the calculation of the FR and cross-section ratio (CSR), HRNS images and video sequences of the site of neuromatous change, the unaffected part of the nerve (> 5 cm proximal to the lesion), and the contralateral nerve, were captured in transverse view for each patient. Additionally, video sequences in color Doppler mode were recorded in transverse view for the calculation of the vascular ratio (VR) with constant gain settings for each patient, respectively. We included a control group of n = 4 healthy volunteers (2 females, 2 males, mean age 47 years) and the VR was calculated in triplicates at defined locations between shoulder and wrist for the ulnar, median, and radial nerve. For inclusion of the nerves’ target muscle status, the color Doppler based muscle perfusion ratio (MPR) was calculated based on transverse images and video sequences of the denervated and contralateral muscle. For each patient, six images of the affected and unaffected nerve and muscle were analyzed.

### Image analysis

**Figure 1:**
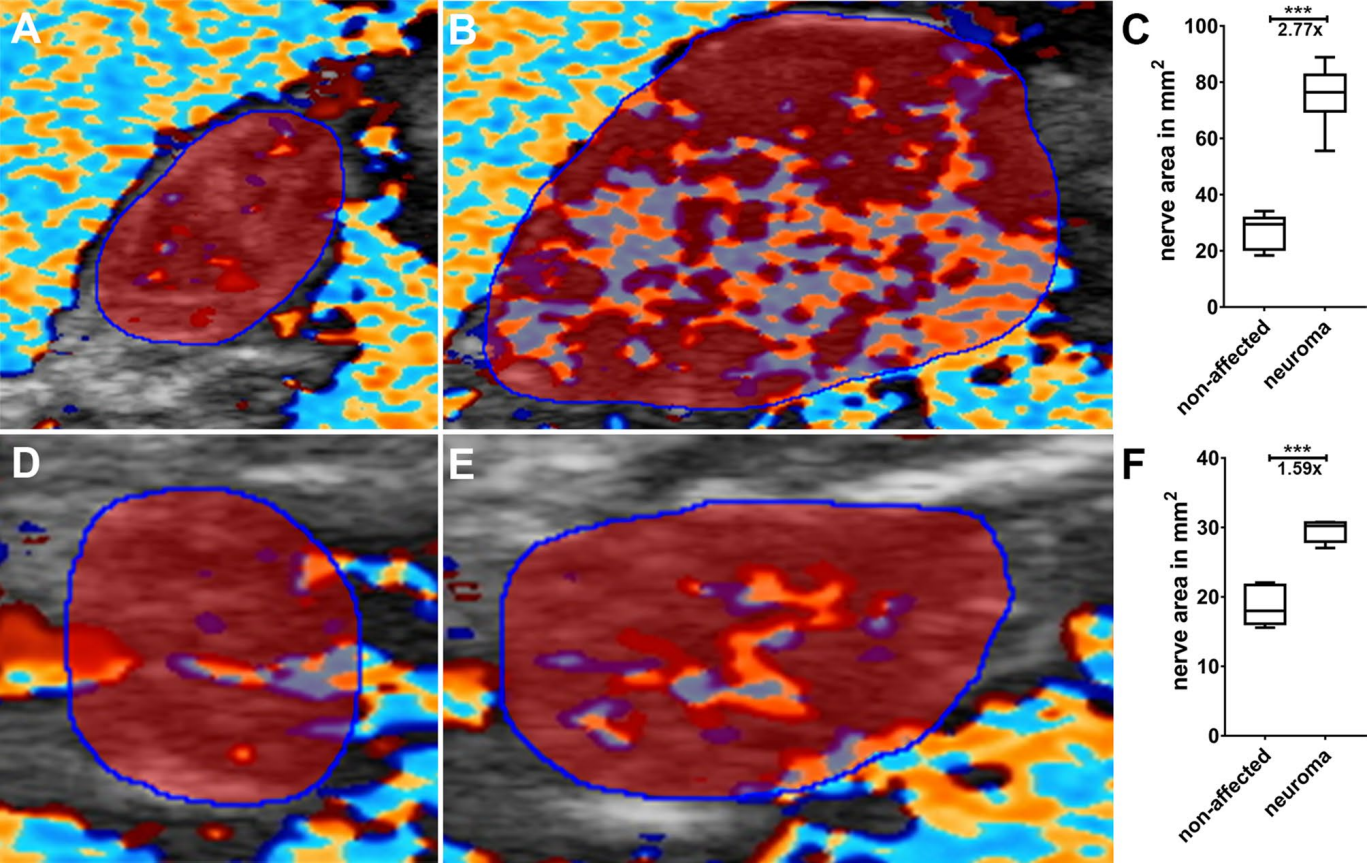
Exemplary quantification of the cross-section area (labeled in red) of the intact nerve portion (**A**,**D**) and neuroma (**B**,**E**). The cross-section area is significantly increased compared to the intact nerve portion (**C**,**F**). The CSR was calculated based on six images for the affected and unaffected nerve portion.

The calculation of the FR, CSR, VR, and MPR was carried out using the scientific image processing program Fiji^4^. The FR was measured as described^2^. In brief, B-mode neurosonographs (transverse view) were converted to 8-bit images, inverted, and the contrast enhanced using background subtraction. Within a manually selected region of interest (ROI) covering the nerve in the transverse view image, hypoechogenic nerve fascicles were selected by automatic thresholding using the “MaxEntropy” threshold. Subsequently, the FR was calculated by measuring hypoechogenic area fraction. Additionally, the CSR was calculated by measuring the cross-section area of affected and unaffected nerve portions. Therefore, the number of pixels within the ROIs were measured, and the CSR was calculated based on the mean values for each patient (see Fig. 1). For calculation of the cross-section area (CSA) in mm^2^, the pixel size was specified as 0.004017 mm^2^.

**Figure 2:**
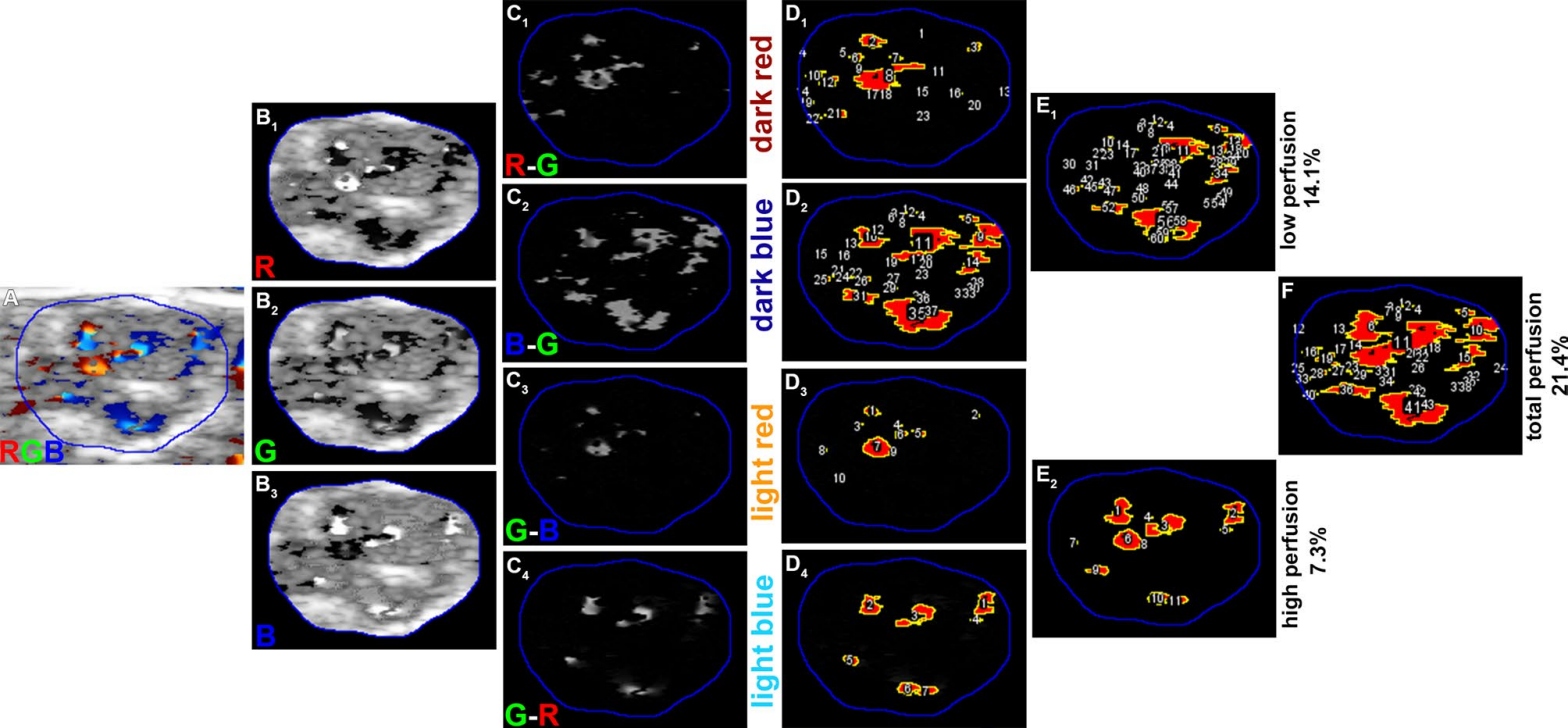
Exemplary analysis of color Doppler neurosonographs and subsequent signal selection. The color Doppler image (**A**) was split into the red, green and blue channel (**B**) and image subtraction steps were performed (**C**) followed by subsequent signal selection via “MaxEntropy”-thresholding and signal selection (**D**). Since the direction of blood flow is not analyzed, the low velocity perfusion signals (**D**_**1**_,**D**_**2**_) as well as the high velocity perfusion signals (**D**_**3**_,**D**_**4**_) were merged via maximum projections, resulting in direction-independent low velocity perfusion signals (**E**_**1**_) and high velocity perfusion signals (**E**_**2**_). For quantification of the total perfusion, these signals were merged via maximum projections respectively (**F**).

**Figure 3:**
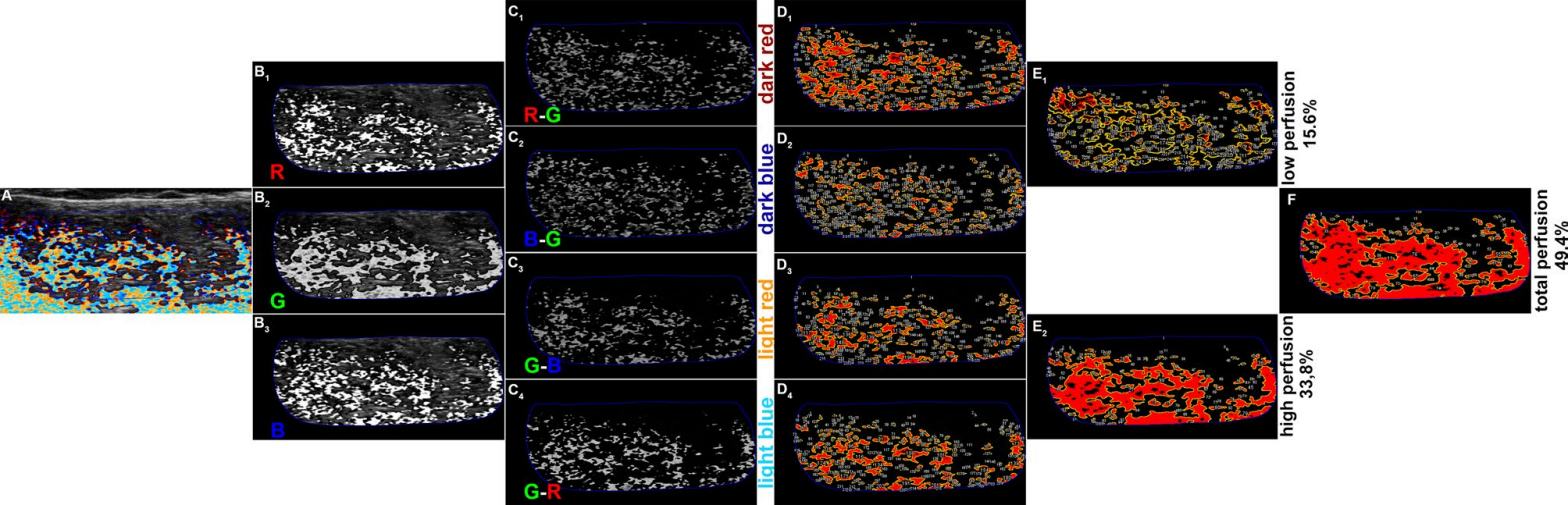
Analysis of color doppler myosonographs (**A**) performed by splitting of the channels (**B**), image subtraction steps (**C**) and signal selection of low and high velocity signals for both directions of blood perfusion (**D**). By creating maximum projections, direction-independent signals were achieved, but since the low velocity perfusion signal includes high velocity perfusion signal (see Supplemental Fig. S1D), the low velocity signal was corrected by subtraction of the high-velocity perfusion signal (**E**_**1**_). Subsequently, the corrected low velocity perfusion signal (**E**_**1**_) and the high velocity perfusion signal (**E**_**2**_) were merged in a maximum projection to gain the signal for the total blood perfusion of the muscle (**F**).

For the quantitative analysis of color Doppler sonographs, representative images were selected from the video sequences in the RGB color spectrum and the nerve portion was manually selected as ROI. Next, the image was split into the red, green and blue channel and areas of high and low velocity blood perfusion were calculated by image subtraction (light blue (high velocity flow away from the transducer): green—red; dark blue (low velocity flow away from the transducer): blue—green; light red (high velocity flow in direction of the transducer): green—blue; dark red (low velocity flow in direction of the transducer): red—green). To extract the low perfusion signal, the high perfusion signal was subtracted, as the initial low perfusion signal includes the signal for the high velocity perfusion (for the not subtracted low perfusion signal see Supplemental Fig. S1). As the direction of blood flow is not analyzed, the images representing high velocity perfusion (light blue and light red) and slow velocity perfusion (dark blue and dark red) were merged as maximum projections. For analysis of the total perfusion, the images of the high and low velocity perfusion were merged respectively. Signals were binarized by automatic thresholding, utilizing the MaxEntropy threshold^5^ for subsequent selection of the signals. The signal was selected by particle analyzer function, measuring the signal area. Subsequently, the VR was calculated as the ratio of the color Doppler signal compared to the nerve area. Analogous to the VR (see Fig. 2), the MPR was calculated based on myosonographs (Fig. 3).

### Statistical analysis

The statistical analysis was carried out using GraphPad Prism 7.00 (GraphPad Software, San Diego, CA, USA). In case the data passed the D’Agostino & Pearson or Shapiro–Wilk test for a Gaussian distribution, the groups were tested for statistical significance by a one-way ANOVA followed by a Tukeys multiple comparison test. For non-parametric testing, the Mann–Whitney or Kruskal–Wallis test (with Dunns multiple comparison test) was conducted. The applied test is stated in the respective figure legends. Levels of statistical significance were set to P-values of p ≤ 0.05 (*), p ≤ 0.01 (**), p ≤ 0.001 (***) for all analysis. Correlation analysis was carried out using the Pearson correlation coefficient.

## Results

### The FR is significantly increased in patients’ neuroma

**Figure 4:**
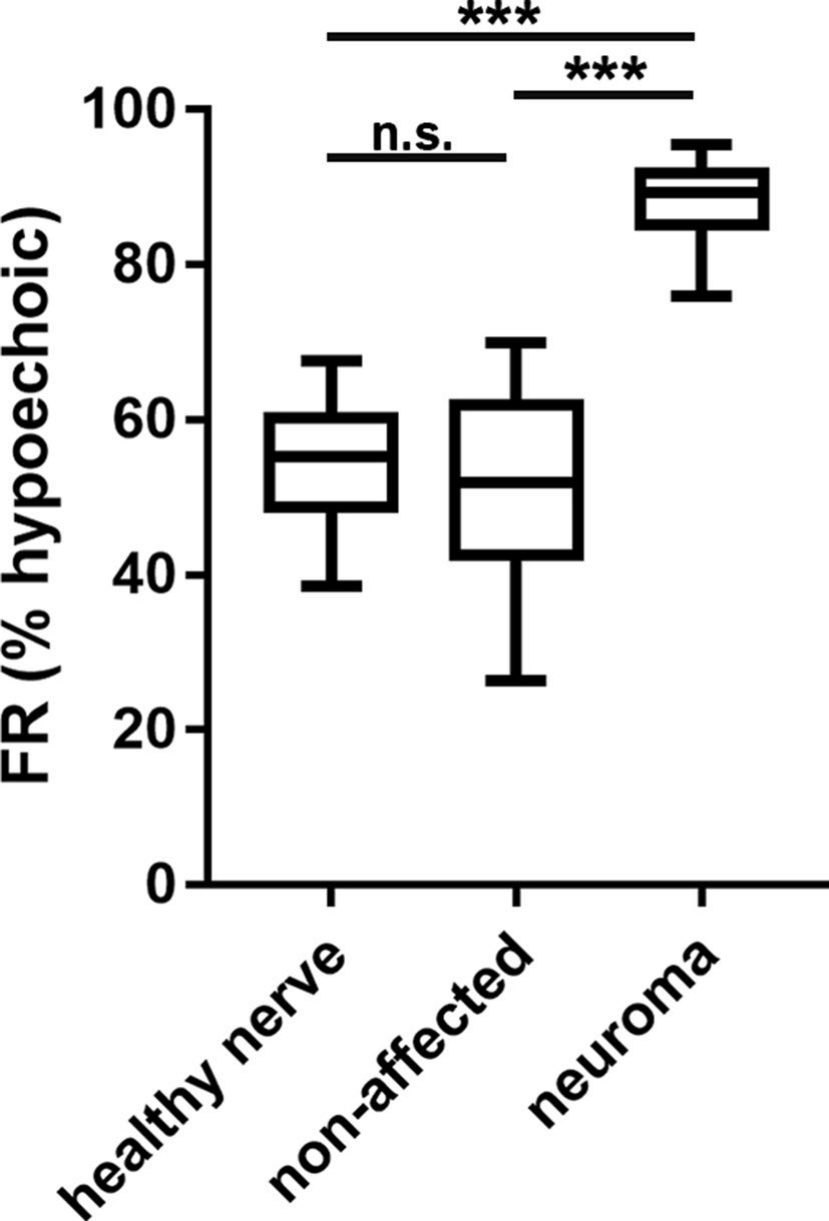
The FR is significantly increased in neuromas compared to the patients' non-affected nerve portions as well as to the volunteers’ healthy control nerves. 1-way ANOVA with Tukey multiple comparison test.

Calculation of the FR resulted in a significantly (p < 0.0001) increased FR for the neuromas (mean FR 87.87%), compared to the non-affected nerve for all 9 included patients (mean FR 50.66%) as well as the healthy volunteers’ FR (mean 54.31%; see Fig. 4). In contrast, the FR of the non-affected patients’ nerves and the FR of healthy volunteers’ nerves was not significantly altered (p > 0.099).

### Image processing leads to reproducible detection of blood flow in color Doppler sonographs of healthy and lesioned nerves

**Figure 5:**
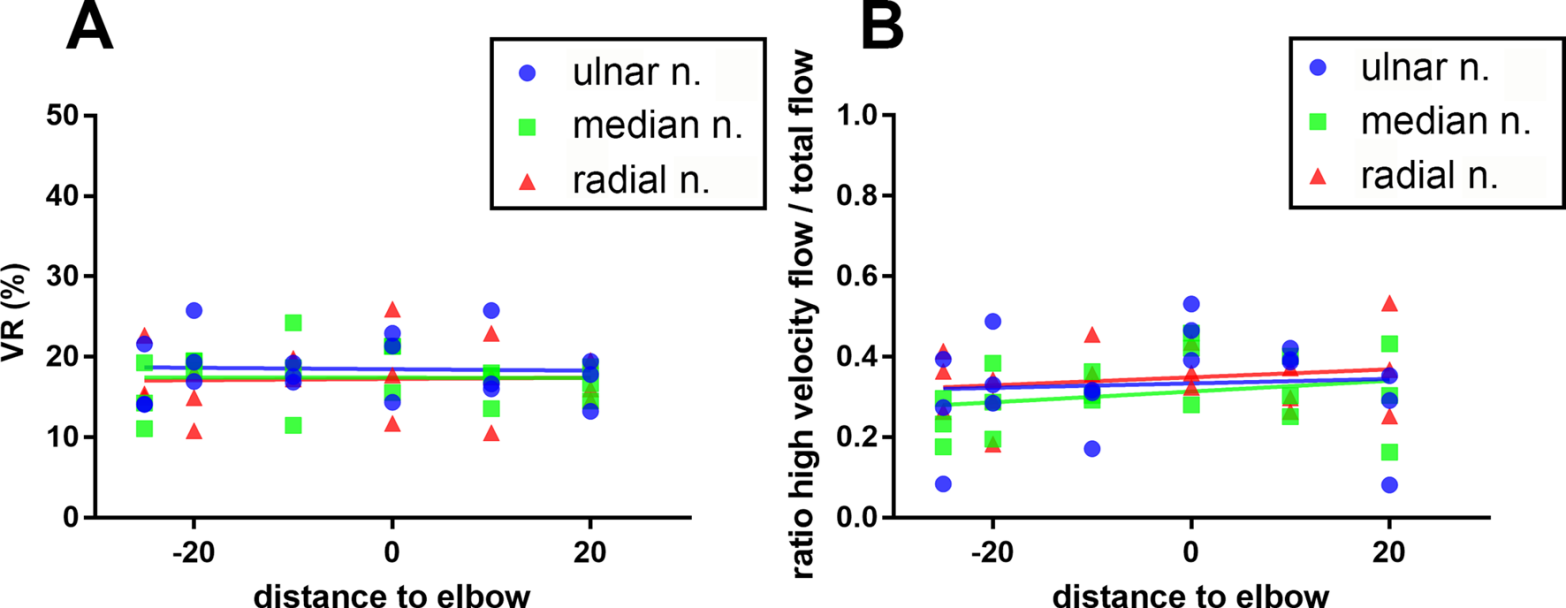
Longitudinal progression of the VR (**A**) and portion of high velocity blood flow (**B**) from the shoulder (negative values) to the wrist (positive values) for the ulnar, median and radial nerve of three healthy volunteers. The slopes of the trendlines do not differ significantly between the ulnar, median and radial nerve, and do not differ from zero for (**A**) and (**B**) respectively.

A comparable calculation of the VR is based on a constant VR, independent of the nerve and the location. The analysis of the VR in the control group confirmed a constant VR (total mean 17.72%, range 10.55–25.94%) for the different sites as well as for the different nerves (mean ulnar 18.51%, mean median 17.43%, mean radial 17.23%). The trendlines for the ulnar, median and radial nerve neither differed significantly, nor was the slope significantly different from zero (Fig. 5A). Moreover, the analysis of the proportion of high velocity blood flow resulted in a mean ratio of 0.328 (ulnar 0.331, median 0.308, radial 0.345) and in no significant difference between the trendlines determined for the ulnar, median and radial nerve, with a slope of no significant difference from zero (Fig. 5B).

### The VR is significantly increased in human neuroma

**Figure 6:**
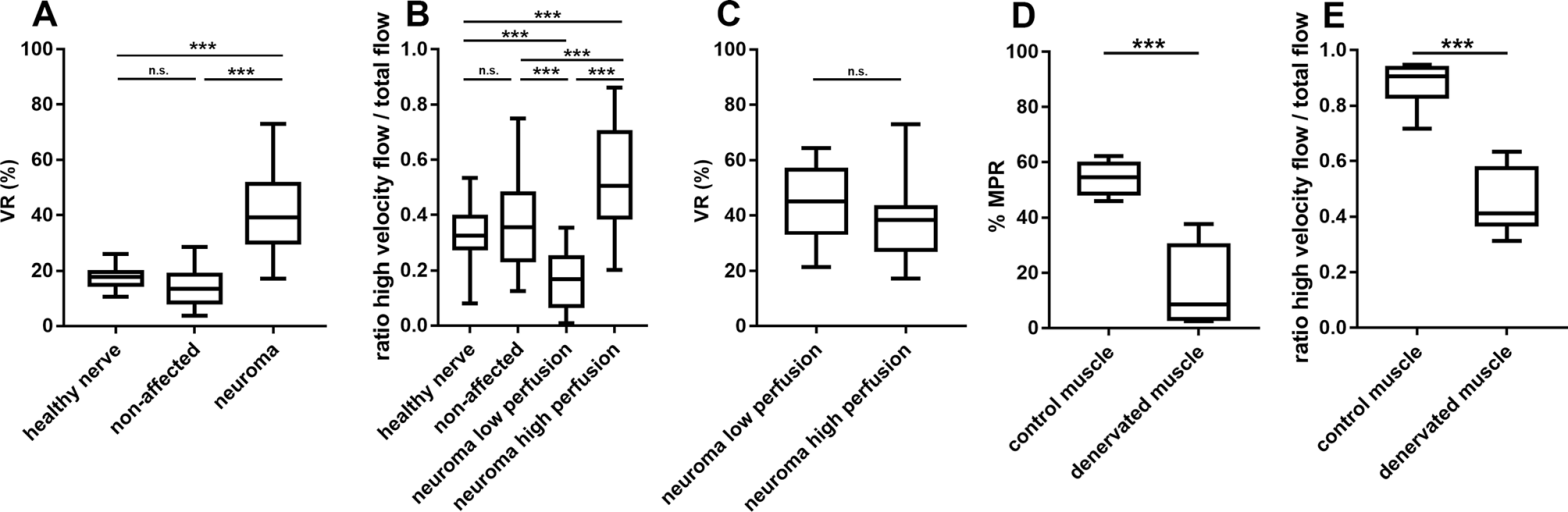
Assessment of VRs and MPRs for intact versus injured nerve and muscle. (**A**) The VRs determined for the neuromas are significantly increased, while the VRs for the healthy nerves and the patients’ unaffected nerve portions show no significant difference. (**B**) The proportion of high velocity blood flow is significantly reduced in the group of low perfusion neuromas, while it is significantly increased in the high perfusion neuromas when compared to the healthy nerve and non-affected nerve portions. Although the rate of high velocity blood flow differs in the high- and low perfusion neuromas, the VRs of both groups do not differ (**C**). The MPRs determined for the denervated muscle are significantly decreased compared to the intact contralateral control muscles (**D**). Moreover, the portion of high velocity blood flow is significantly decreased in the denervated muscle compared to the control (**E**). (**A**,**B**) 1-way ANOVA with Tukey multiple comparison test; (**C**) unpaired t-test; (**D**,**E**) Mann–Whitney test.

The calculation of the VR in neuromas of 9 patients resulted in a significantly increased VR for the neuromas with a mean VR of 43.26%, compared to the VR of the healthy volunteers’ nerves (mean 17.72%) and the patients’ non-affected nerve portions (mean 14.14%; Fig. 6A). A significant difference between the healthy nerve and the unaffected nerve portion was not detected. Next, we analyzed the proportion of high velocity blood flow in relation to the total perfusion. We found two types of neuroma: type 1 with a reduced high velocity perfusion, and type 2 with an increased high velocity perfusion compared to the healthy and unaffected nerves (Fig. 6B). Although the proportion of high velocity flow is significantly different from the control nerves for high and low perfused neuromas, the VR is constant for both types (see Fig. 6C). Thus, the area of the color Doppler signal is significantly increased for the low perfusion neuroma (mean 45.16%) as well as the high perfusion neuroma (mean 42.12%) compared to the controls (mean non-affected nerve 14.14%; mean healthy control 17.72%), but the high velocity blood flow color Doppler signal is significantly reduced in the low perfusion neuroma (mean 0.161), while it is significantly increased in the high perfusion neuroma (mean 0.560) compared to the controls (mean unaffected nerve 0.390; mean healthy control 0.328).

For the painful neuromas (2 of the 9 assessed neuromas) we found a mean VR of 30.17%, which was significantly increased compared to the VR of the non-affected nerve 14.14% and healthy control 17.72% (p ≤ 0.0001, Kruskal Wallis with Dunns multiple comparison test). There was no statistical difference of the VR of painful neuromas (mean 30.17%) compared to non-painful neuromas (mean 43.37%).

### The CSR is highly variable in the human neuroma

For the quantitative analysis of the neuromatous enlargement of the nerve, the nerve cross section area (in mm^2^) was measured in the neuroma and the non-affected nerve portion. Subsequently, the enlargement-factor was calculated based on the mean of the measurements for each patient, resulting in a mean 2.20-fold increase in nerve size for the neuromas compared to the non-affected nerve portions. However, the CSR ranged from 1.02 to 3.40 and thus, varied widely between the assessed neuromas.

### The MPR is decreased in the denervated muscle

We quantified the perfusion of the denervated muscle and the contralateral control muscle and calculated the MPR based on the ratio of color Doppler signal and the total muscle area. For the intact contralateral muscle, a mean MPR of 54.15% was calculated, whereas the denervated muscles revealed a significantly reduced mean MPR of 14.22% (Fig. 6D). Moreover, the proportion of high velocity blood flow is significantly reduced in the denervated muscle (mean 0.454) compared to the intact contralateral muscle (mean 0.875, Fig. 6E).

### Correlation of VR, FR and CSA

**Figure 7:**
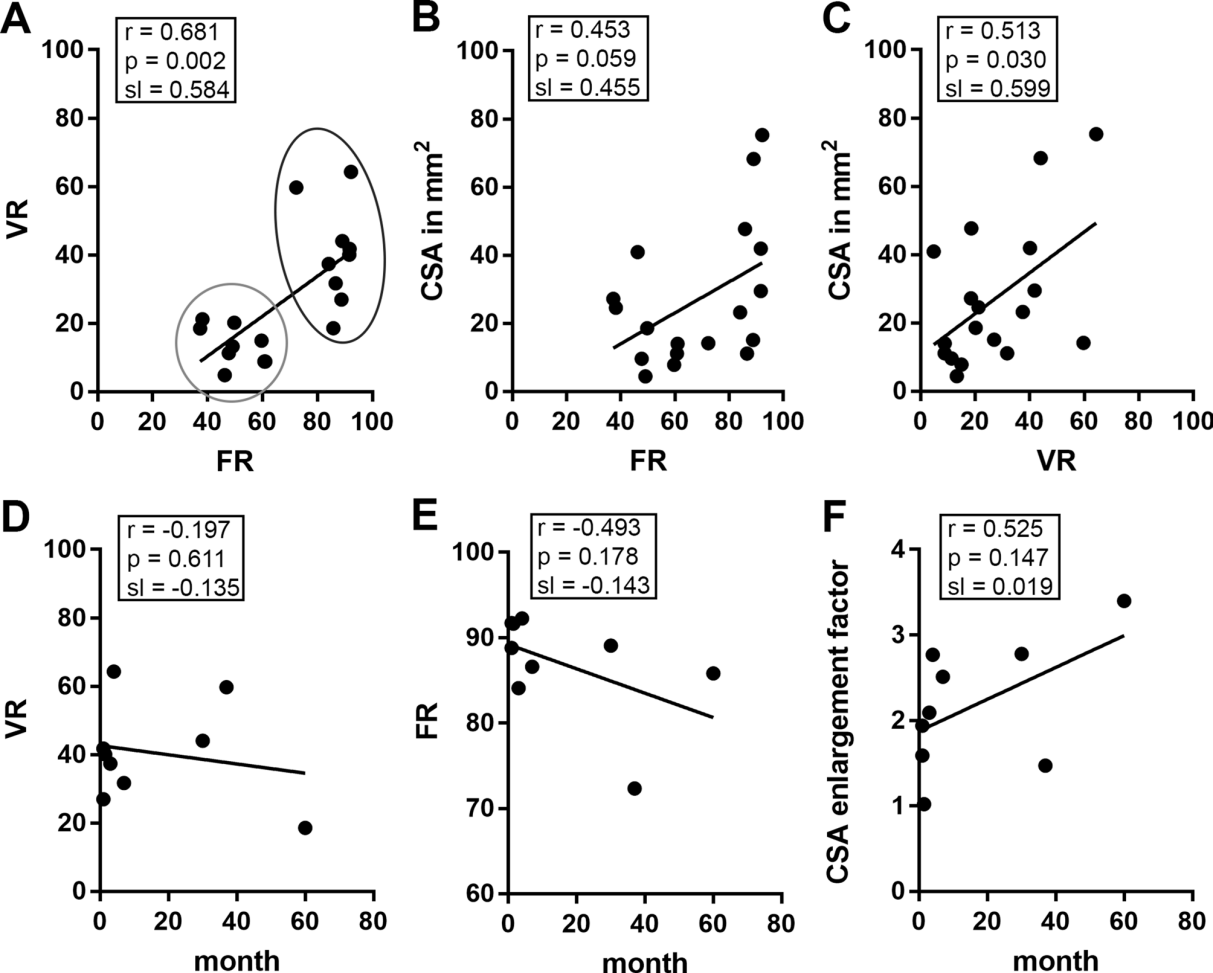
Pearson correlation analysis of the determined FRs, VRs and CSAs showed a significant positive correlation for the FRs and VRs (**A**). Values of the non-affected nerves are encircled in light grey and the neuroma-group is labeled in dark grey. The correlation between FRs and CSAs is not significant but shows a strong trend (**B**), while the VRs and CSAs again revealed significant correlation (**C**). No significant correlation was found between the post-trauma time (neuroma development) and the VRs (**D**), FRs (**E**), as well as the relative CSA enlargement (**F**).

In a further step, we tested the variables VR and CSA for correlation with the FR. The FR and VR showed a strong correlation with a correlation coefficient of 0.681 (p = 0.002, see Fig. 7A). In the non-affected nerve portion, low FR- and VR-values were revealed by quantitative analysis (see Fig. 7A, light grey circle), while high FR values were associated with increased VR values in the neuromatous nerve portions (see Fig. 7A, dark grey circle). In contrast, correlation of the FR and VR with CSA appeared weak. Due to large variations in the cross-section area within the group of neuromas and non-affected nerves, FR and CSA showed a trend for a correlation (r = 0.453) without being significant (p = 0.059, see Fig. 7B) while VR and CSA correlated significantly (r = 0.513, p = 0.03; see Fig. 7C). Correlation analyses of the VR, FR and CSA with neuroma age (see Fig. 7D–F) revealed no significant correlations.

## Discussion

In assessment of nerve lesions and post-operative regeneration HRNS has become a highly valuable tool. However, it cannot provide direct visualization of axonal regrowth due to limited spatial resolution^1^. The establishment of new parameters, such as the “fascicular ratio” (FR)^5,6^ has facilitated a quantitative assessment of traumatic nerve lesions. As shown in a recent study by Heinen et al. (2018), FR allows for a reliable distinction between healthy and lesioned nerves and indirect tracking of regeneration. The FR of recovering nerves significantly changed back from initial pathologic values to physiological values. This change coincided in 71% of the affected patients with clinical and/or electrophysiological reinnervation. In search for further regeneration parameters in HRNS, we have encountered a study by Cattin et al. (2015). They postulate a major role of the vascular system for regeneration by providing guidance for Schwann cells and axons in rodents. To analyze this finding in patients, we examined the vascularization in nerve trauma using Doppler HRNS.

For the quantitative analysis of blood flow in color Doppler sonographs, a new method based on the scientific image processing software Fiji was established. The color Doppler signals were extracted by image calculation and subsequent signal selection of high and low perfusion signals by automatic thresholding. This procedure provides reproducible and reliable information on artificial data as well as patient derived color Doppler sonographs of nerve and muscle tissue. In this study, we subsequently evaluated the total perfusion and the proportion of high velocity blood flow, based on the extracted color Doppler signals of neuro- and myosonographs.

Alterations of vascularization in other nerve lesions have been described using HRNS. In carpal tunnel syndrome (CTS), Akcar et al. (2010) reported on 50% of the CTS patients showing blood flow signal when using power Doppler. In unaffected controls, no intraneural blood flow could be found. They explained this phenomenon by the assumed pathophysiology of an entrapment syndrome with venous congestion, nerve edema and impairment of the arterio-venous supply. CSA was elevated in CTS with a cut-off at 11 mm^2^^7^. Ghasemi-Esfe et al. (2011) stated that color and power Doppler had similar sensitivities and specificities compared to electrodiagnostic measures. Moreover, they propose that the severity of CTS (determined by electrophysiology) could be assessed by Doppler sonography^8^. Since excessive scar tissue within and around the lesioned nerve area has a constricting effect on the blood vessels, the perfusion velocity at the constriction-site might be increased. This may result in a reduced perfusion velocity of the neuroma, which we could observe in the low perfusion neuroma (see Fig. 6). In contrast to other authors^9–12^ we propose that assessing CSA alone is insufficient for proper diagnosis of neuromatous change, since CSA was not increased in all examined neuromas (Fig. 7). However, CSA can be used as an indicative factor, but should be enhanced by determination of FR and/or VR.

Vascular changes in HRNS cannot solely be observed in compression neuropathies. In 2009, Shankar et al. examined n = 1 patient with a painful stump neuroma and described the changes of vascularity at different examinations. Periods of heavy pain were associated with extensive vascularity, whereas during low or no pain a complete absence of vascularity was observed. They assume that alpha-adrenergic receptors in the vasa nervorum may mediate this vascular response. The authors also addressed the implication of the surrounding scar tissue in nerve’s vascularity^13^. In our limited experience, all n = 2 patients with a painful neuroma showed a higher vascularity within the neuroma (mean VR in painful neuroma 30.17%), compared to non-affected (mean VR 14.14%) and healthy control nerves (mean VR 17.72%). However, we do not have any sequential imaging data.

There are only few reports on the usage of Doppler ultrasound in traumatic nerve lesions^14–16^. In 2018, Aranyi et al. published a paper dealing with penetrating nerve lesions in n = 30 patients (n = 34 lesions) including neuromas in continuity, stump neuromas and suture sites. All patients exhibited hypervascularization in the nerve portion proximal to the lesion, but not in the lesion itself^17^. This is contrary to our observations. However, Aranyi et al. (2018) found no correlation between the degree of vascularization (assessed by percentage of vascular signals in a hand-selected ROI of a transverse scan), age, lesion size and clinical reinnervation. The latter goes along with our results. They calculated the percentage of Doppler signal pixels within the hand-selected area reaching from 0.3–19.1%. We additionally assessed the velocity of blood perfusion, which resulted in the differentiation of two neuroma types: the high and low velocity perfused neuroma. While the high velocity perfused neuroma is presumably associated with an increased capillary vessel formation within the neuroma, a vascular constriction due to extensive scar formation proximal to the neuroma might lead to low velocity perfused neuromas. However, postoperative data is required to correlate the outcome with the neuroma perfusion status.

Furthermore, we assessed the vascular changes within the effector muscle. To the best of our knowledge, this is the first quantitative assessment of the denervated target muscle perfusion following traumatic nerve injury. MPR was calculated using the same algorithm as for the VR. We found a significantly decreased perfusion of the denervated muscle compared to the contralateral healthy control muscle. Moreover, the portion of high velocity blood flow was significantly decreased. In contrast to our findings, experiments in rabbits suggest an increased perfusion of denervated muscles within the first 9 days following denervation^18^. However, our assessment was conducted at least one month after trauma impeding insight into acute phase alterations. The MPR and the proportion of the muscles high velocity blood flow could indirectly reflect muscle denervation and nerve damage. However, it should be kept in mind, that not only the sensorimotor but also the vegetative fibers are impaired in nerve trauma.

A clear limitation of our study is the low number of patients and the lack of postoperative data. Thus, it is not clear, whether the VR or MPR represent potential prognostic tools for predicting the outcome after treatment. Assuming the proposed mechanisms of blood vessel formation preceding the re-growing axons and Schwann cells^3^, vascularization should change within the affected nerve as well as the muscle during regeneration. Based on these findings of Cattin et al. (2015), we would expect to detect vessels to precede the axonal growth cone.

## Conclusion

In peripheral nerve trauma, Doppler enhanced HRNS facilitates visualization of vascularity in the affected nerve and the target muscle. In combination with the more morphologic FR and CSR, the MPR and VR could give insight into the vascular alterations. Moreover, data acquisition is feasible, and the parameters are straightforward to calculate. Of course, further studies are mandatory.

## Supplementary Information

Supplementary Information.

## References

[1] Koenig RW (2009). High-resolution ultrasonography in evaluating peripheral nerve entrapment and trauma. Neurosurg. Focus.

[2] Heinen C (2019). Fascicular ratio pilot study: High-resolution neurosonography: A possible tool for quantitative assessment of traumatic peripheral nerve lesions before and after nerve surgery. Neurosurgery.

[3] Cattin A-L (2015). Macrophage-induced blood vessels guide schwann cell-mediated regeneration of peripheral nerves. Cell.

[4] Schindelin J (2012). Fiji: An open-source platform for biological-image analysis. Nat. Methods.

[5] Boom J, Visser LH (2012). Quantitative assessment of nerve echogenicity: Comparison of methods for evaluating nerve echogenicity in ulnar neuropathy at the elbow. Clin. Neurophysiol..

[6] Tagliafico AS, Tagliafico G (2014). Fascicular ratio: A new parameter to evaluate peripheral nerve pathology on magnetic resonance imaging. Medicine.

[7] Akcar N, Özkan S, Mehmetoglu Ö, Calisir C, Adapinar B (2010). Value of power Doppler and Gray-scale US in the diagnosis of carpal tunnel syndrome: Contribution of cross-sectional area just before the tunnel inlet as compared with the cross-sectional area at the tunnel. Korean J. Radiol..

[8] Ghasemi-Esfe AR (2011). Color and power Doppler US for diagnosing carpal tunnel syndrome and determining its severity: A quantitative image processing method. Radiology.

[9] Mallouhi A, Pültzl P, Trieb T, Piza H, Bodner G (2006). Predictors of carpal tunnel syndrome: Accuracy of gray-scale and color Doppler sonography. Am. J. Roentgenol..

[10] Joy V, Therimadasamy AK, Chan YC, Wilder-Smith EP (2011). Combined Doppler and B-mode sonography in carpal tunnel syndrome. J. Neurol. Sci..

[11] Osada R, Zukawa M, Kimura T (2018). Ultasonographic indicators of carpal tunnel syndrome demonstrate reversibility following carpal tunnel release. Radiol. Diagn. Imaging.

[12] Kotb MA, Bedewi MA, Aldossary NM, Mahmoud G, Naguib MF (2018). Sonographic assessment of carpal tunnel syndrome in diabetic patients with and without polyneuropathy. Medicine.

[13] Shankar H (2009). Ultrasound demonstration of vascularity changes with changes in pain perception in a stump neuroma. Clin. J. Pain.

[14] Frijlink DW, Brekelmans GJF, Visser LH (2013). Increased nerve vascularization detected by color doppler sonography in patients with ulnar neuropathy at the elbow indicates axonal damage. Muscle Nerve.

[15] Chiou H-J (2010). Soft-tissue tumor differentiation using 3D power doppler ultrasonography with echo-contrast medium injection. J. Chin. Med. Assoc..

[16] Li X, Li JW, Ho AM-H, Karmakar MK (2015). Age-related differences in the quantitative echo texture of the median nerve. J. Ultrasound Med..

[17] Arányi Z, Csillik A, Dévay K, Rosero M (2018). Ultrasonographic demonstration of intraneural neovascularization after penetrating nerve injury: Neovascularization after nerve injury. Muscle Nerve.

[18] Goyault G (2012). Diffusion-weighted MRI, dynamic susceptibility contrast MRI and ultrasound perfusion quantification of denervated muscle in rabbits. Skelet. Radiol..

