# Supplementary material for: Quantitative assessment of intraneural vascular alterations in peripheral nerve trauma using high-resolution neurosonography: technical note
Source: Sci Rep. 2021 Jun 25;11:13320. doi: 10.1038/s41598-021-92643-9 (PMC8233368; doi:10.1038/s41598-021-92643-9)
Supplement: Supplementary file 1 — Supplementary Information. [file 41598_2021_92643_MOESM1_ESM.docx]

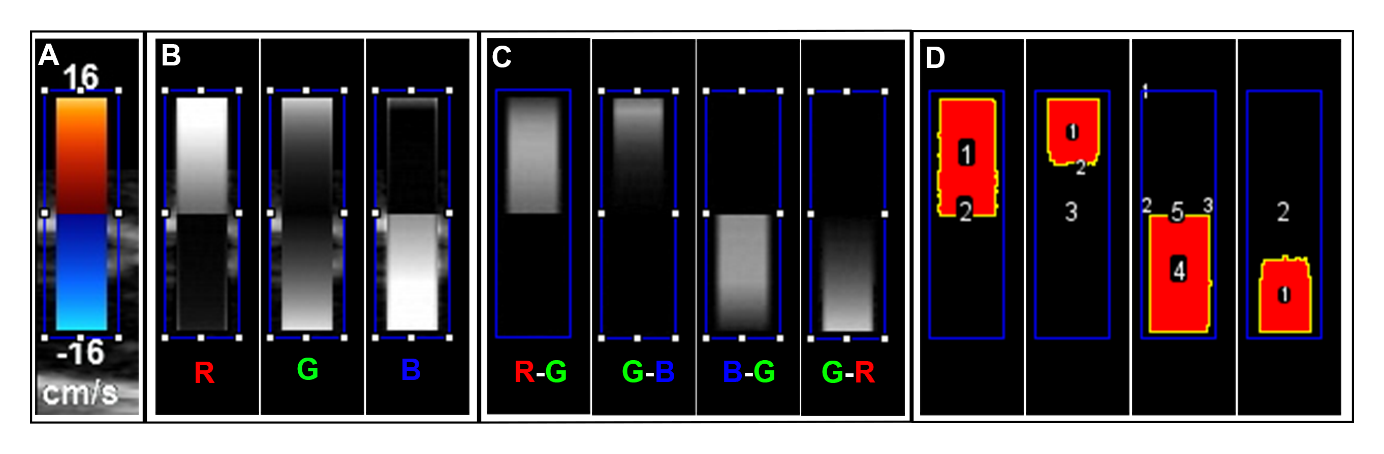


Supplemental Figure 1: Overview of color Doppler image processing based on artificial data. Color Doppler images in the RGB color space (A) were split into the red, green and blue channel (B). Image subtraction leads to separation of dark blue and dark red, as well as light blue and light red signals (C). These signals were extracted using the “MaxEntropy”-threshold with subsequent selection of the signal area by the “particle analyzer”-function of Fiji (D).
